# The effect of surgical suture material on osteoclast generation and implant-loosening

**DOI:** 10.7150/ijms.50270

**Published:** 2021-01-01

**Authors:** Ulrike Dapunt, Birgit Prior, Jan Philippe Kretzer, Gertrud Maria Hänsch, Matthias Martin Gaida

**Affiliations:** 1 Center for Orthopaedics, Trauma Surgery and Spinal Cord Injury, Heidelberg University Hospital, Schlierbacher Landstrasse, Heidelberg, Germany.; 2 Department of Anesthesiology, Heidelberg University Hospital, Heidelberg.; 3 Laboratory of Biomechanics and Implant Research, Center for Orthopaedics, Trauma Surgery and Spinal Cord Injury, Heidelberg University Hospital, Heidelberg, Germany.; 4 Institute of Immunology, Heidelberg University. Heidelberg, Germany.; 5 Institute of Pathology, Universitätsmedizin der Johannes Gutenberg Universität Mainz, Germany.

**Keywords:** Osteoclast, osteolysis, surgical suture material, implant-associated infection, Interleukin-8

## Abstract

**Background:** Implant loosening - either infectious or aseptic- is a still a major complication in the field of orthopaedic surgery. In both cases, a pro-inflammatory peri-prosthetic environment is generated by the immune system - either triggered by bacteria or by implant wear particles - which leads to osteoclast differentiation and osteolysis. Since infectious cases in particular often require multiple revision surgeries, we wondered whether commonly used surgical suture material may also activate the immune system and thus contribute to loss of bone substance by generation of osteoclasts.

**Methods:** Tissue samples from patients suffering from infectious implant loosening were collected intraoperatively and presence of osteoclasts was evaluated by histopathology and immunohistochemistry. Further on, human monocytes were isolated from peripheral blood and stimulated with surgical suture material. Cell supernatant samples were collected and ELISA analysis for the pro-inflammatory cytokine IL-8 was performed. These experiments were additionally carried out on ivory slices to demonstrate functionality of osteoclasts. Whole blood samples were incubated with surgical suture material and up-regulation of activation-associated cell surface markers CD11b and CD66b on neutrophils was evaluated by flow cytofluorometry analysis.

**Results:** We were able to demonstrate that multinucleated giant cells form in direct vicinity to surgical suture material. These cells stained positive for cathepsin K, which is a typical protease found in osteoclasts. By *in vitro* analysis, we were able to show that monocytes differentiated into osteoclasts when stimulated with surgical suture material. Resorption pits on ivory slices provided proof that the osteoclasts were functional. Release of IL-8 into cell supernatant was increased after stimulation with suture material and was further enhanced if minor amounts of bacterial lipoteichoic acid (LTA) were added. Neutrophils were also activated by surgical suture material and up-regulation of CD11b and CD66b could be seen.

**Conclusion:** We were able to demonstrate that surgical suture material induces a pro-inflammatory response of immune cells which leads to osteoclast differentiation, in particular in combination with bacterial infection. In conclusion, surgical suture material -aside from bacteria and implant wear particles- is a contributing factor in implant loosening.

## Introduction

Even though total joint replacement of the hip and knee is considered one of the most successful surgical procedures in the field of orthopaedics, infectious or aseptic implant loosening is still a severe complication 1-4.

In the case of infections, bacteria form biofilm colonies on an implant surface, which makes them more difficult to detect and to treat. Patients often suffer from a prolonged treatment course, which usually requires extensive antibiotic treatment and multiple revision surgeries; possibly including implant-exchange due to loosening 5-7.

In our previous work, we were able to demonstrate that the pro-inflammatory peri-prosthetic microenvironment, which is generated by a persistent immune response, leads to increased osteoclast generation, bone degradation and in consequence implant loosening 8-10.

We were able to characterize the cellular infiltrate and identify various pro-inflammatory cytokines that are enhanced in these cases and that stimulate osteoclast generation 11.

In the case of so-called aseptic implant loosening where no bacteria are detected, osteolysis is presumably induced by an inflammatory reaction towards implant wear particles 4. We also evaluated aseptic cases in our previous studies and were able to show, that the pro-inflammatory peri-prosthetic environment is very similar to that found in infection 11, 12. However, the response was less pronounced for the relevant cytokines. This finding corresponds with the clinical observation that patients with aseptic loosening usually become symptomatic later on compared to infectious cases, but the inflammatory response will ultimately also lead to generation of osteoclasts and hence implant loosening.

On the other hand, because biofilm infections are difficult to detect and up to 40% of falsely - negative cases have been reported, the question whether aseptic loosening is truly “aseptic” remains a subject of much debate 13-15. Moreover, it has been shown that endotoxin adherent on orthopaedic wear particles induces significantly higher cytokine production and osteoclast differentiation, indicative of a combination of factors that eventually trigger an enhanced and persistent pro-inflammatory immune response 16, 17.

In this context, we were interested whether surgical suture material - a foreign body material omnipresent in surgical sites - might similarly contribute to osteoclast generation and further on implant loosening. Several reports can be found in literature that support the notion of an inflammatory response directed towards surgical suture material 18-21. Multinucleated giant cells have been detected in direct vicinity to suture material via histopathology 22. Lock and al co-cultivated human monocyte THP-1 cells with seven commonly used suture materials and were able to demonstrate by gene expression and protein secretion analyses that certain sutures in particular induced up-regulation of pro-inflammatory cytokines 23.

Aim of this study was to investigate whether multinucleated giant cells in association with surgical suture material are in fact functional osteoclasts, capable of degrading bone substance and therefore also contributing to implant loosening.

## Materials and Methods

### Patients

Patients (n=5) who underwent revision surgery due to a prosthetic infection with loosening of the implant were included in the study. Diagnosis of loosening of the implant was based on patients' complaints, clinical examination, and examination by conventional X-ray and/or CT scan.

### Collection of tissue samples and histology

From five patients with an infection tissue samples were taken from sites of osteolysis. The study was approved by the local ethics committee of Heidelberg University (No. 206/2005).

The tissue specimens were formalin-fixed, decalcified in ethylenediaminetetraacetic acid (EDTA), and paraffin embedded. Haematoxylin and eosin (H&E) staining was performed. The biopsies were examined, the diagnosis of an acute or chronic osteomyelitis was made, and the cellular infiltrates in particular the osteoclasts were evaluated. The paraffin-embedded tissue sections (3-4 *μ*m) were also used for immunohistochemical analyses. Immunostaining was performed as previously described using the avidin-biotin complex method 24. Prior to antibody incubation, heat pretreatment in citrate buffer (pH 6.1) was performed. As primary antibody, the monoclonal mouse anti cathepsin K (Calbiochem, San Diego, USA) was used.

### Isolation of monocytes

Monocytes were isolated from the peripheral blood of healthy donors (informed consent was obtained and the institutional guidelines were observed). The blood was layered on Polymorphprep (Axis Shield, Oslo, Norway), and the monocyte fraction was recovered. Monocytes were seeded into 24-well dishes (NuncTM, Wiesbaden, Germany) at a concentration of 3×10^6^ per well and selected by adherence after incubation for 2 hours in RPMI containing 1% glutamine, and 1% penicillin/streptomycin (medium and supplements were obtained from Gibco Life Technologies, Darmstadt, Germany). Monocytes were carefully washed to remove any non-adherent cells and 1 mL of RPMI containing 10% fetal calf serum, 1% glutamine, and 1% penicillin/streptomycin (medium and supplements were obtained from Gibco Life Technologies, Darmstadt, Germany) was added to each well.

### ELISA

IL-8 level in cell culture supernatants were determined using commercially available ELISA kits according to the protocol provided by the manufacturer (R&D Systems, Minneapolis, USA).

### Surgical suture material

Coated VICRYL (polyglactin 910) was used for experiments, because this type of suture material was used for deep fascial and subcutaneous layers in patients. Sutures (0,270 g) were shredded using a scalpel and resuspended in 20 mL RPMI containing 10% fetal calf serum and 1% penicillin/streptomycin.

### Generation of osteoclasts

Monocytes were prepared as described above. For stimulation, the following was added: -medium only, -LTA 0,1 µg/mL (Sigma, Munich, Germany), -surgical suture material 300 µg/ml, - LTA 0,1 µg/ml and surgical suture material 300 µg/mL, - M-CSF 25 ng/mL (R&D Systems, Minneapolis, USA) and RANKL 50 ng/mL (PeproTech, Hamburg, Germany) (positive control).

Cultures were incubated at 37°C in 5% CO_2_ for 16 days with a change of medium and removal of nonadherent cells at day 7 and day 14. Cell supernatant samples for IL-8 ELISA were obtained after 48 hours, 7 days and 14 days.

For the follow up differentiation, the cells were fixed with 4% PFA for 15 minutes at 37°C and identified by their morphological appearance as giant cells with multiple nuclei. Binding of FITC-labeled Phalloidin (Sigma-Aldrich; 1 : 20 dilution for 40 minutes) revealed the typical actin ring formation. Nuclei were stained using DAPI (Invitrogen, Oregon, USA), (diluted 1 : 20000 for 5 minutes).

Fluorescence was visualized using a Digital Fluorescence Microscope (Keyence, Neu-Isenburg, Germany).

Functionality of osteoclasts was tested by differentiating monocytes on ivory slices for 16 days, followed by toluidine blue staining and quantification of the resorption pits by light microscopy (ivory was obtained from the Bundesamt für Umweltschutz, Bonn, Germany).

### Functional assay with neutrophils

To heparinized blood, LTA 0, 1 µg, 200 µl of suture material suspension and LTA combined with suture material was added, followed by incubation for 30 min. CD11b and CD66b expression was measured by cytofluorometry. Directly labelled antibody to CD11b (FITC) or CD66b (FITC) (both obtained from Beckman Coulter, Krefeld, Germany) were used and for comparison isotypic mouse IgG - FITC (BD Bioscience Pharmingen, San Jose, CA, USA). The samples were analysed using FACSCalibur (Becton Dickinson, Heidelberg, Germany) and Cell Quest Pro software (Version 4.0.2, Becton Dickinson).

### Statistical Analysis

Differences between groups were calculated using Mann-Whitney test and unpaired t-test using Graph Pad Prism 8.4.1 software. Significance level was determined as *p*<0.05.

## Results

**Figure 1:**
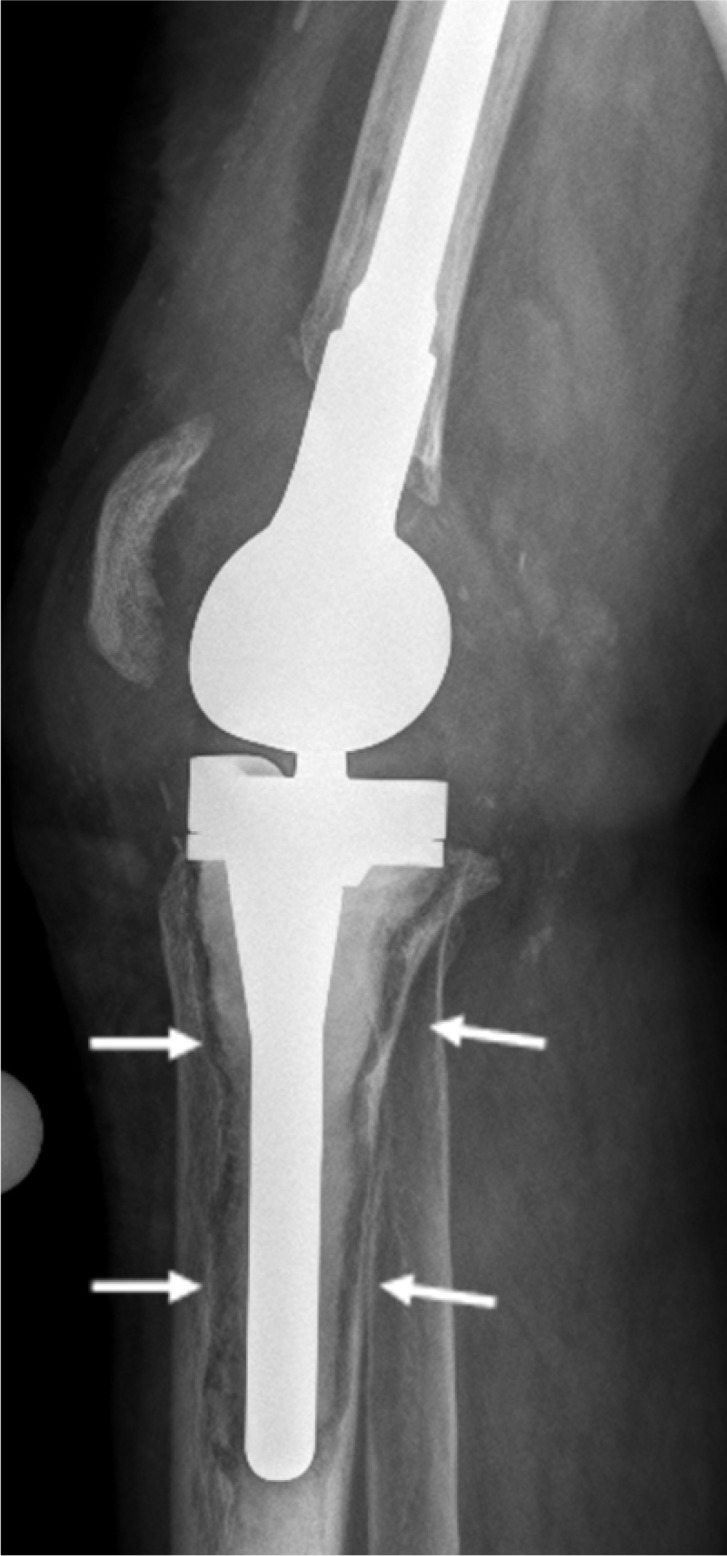
X-ray of a patient suffering from infectious implant loosening of a total knee replacement. Radiolucent lines can be seen particularly around the tibial implant component, indicative of loosening (white arrows).

**Figure 2:**
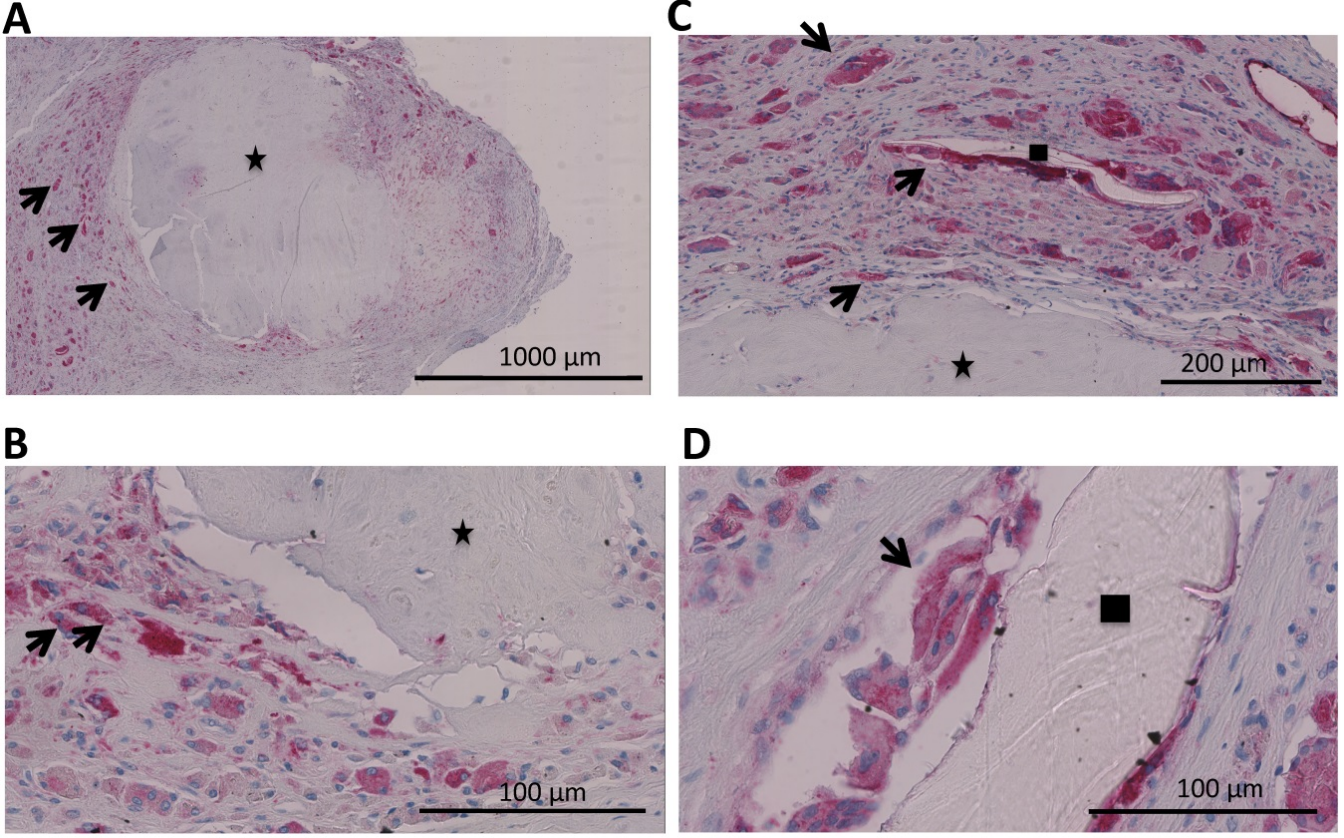
** A-D.** Analysis of a patients' biopsy with prosthetic joint infection. **A,B** Eroded bone (identified by black asterix) is seen and next to it osteoclasts (identified by an antibody for cathepsin K (dark red) and multiple nuclei (blue)) (black arrows). **C,D** Multinucleated giant cells that were positive for cathepsin K (osteoclasts; black arrows) could also be found in direct vicinity to surgical suture material (identified by black square).

Tissue samples were collected intraoperatively from areas of osteolysis from patients suffering from infectious implant loosening (**Figure 1**). Patients' clinical data are shown in **Table 1.** Diagnosis of implant-associated infection was confirmed by histology (>23 neutrophils per 10 high power fields) 25. H&E staining showed multinucleated giant cells in direct vicinity to surgical suture material. Additionally, immunohistochemical staining for cathepsin K, which is characteristic for osteoclasts, was performed and giant cells in association with suture material showed positive results (**Figure 2A-D**).

These results were followed up by *in vitro* analysis. Monocytes were isolated and stimulated with surgical suture material and/or LTA (lipoteichoic acid), the latter a constituent of the cell wall of gram-positive bacteria.

**Figure 3:**
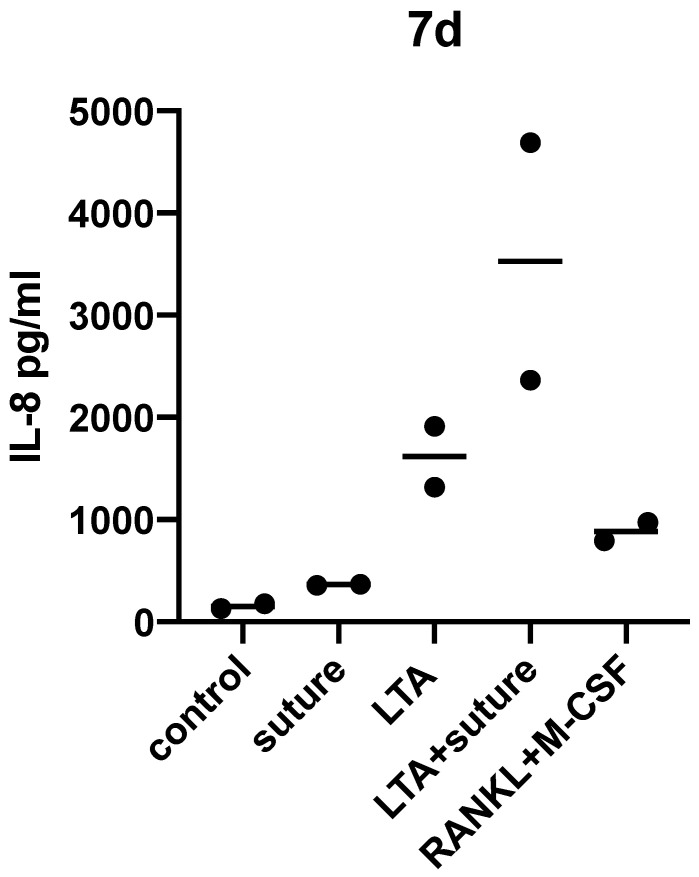
Cell supernatants were collected after 7 days and IL-8 ELISA (pg/ml) was performed. Increased IL-8 release could be detected compared to medium only (control).

Samples of cell supernatant were collected after 48 hours, 7 days and 14 days for IL-8 ELISA analysis (**Table 2; Figure 3**). Experiments were repeated twice and the results after 7 days of incubation are shown.

After 7 days of incubation with surgical suture material, IL-8 release was significantly higher compared to the control sample (*p*=0.0127 as tested by unpaired *t*-test).

The individual response varied greatly between different donors and therefore statistical significance was not reached when comparing LTA and LTA+suture. However, an increased response to LTA+suture could be observed for each donor as shown in **Figure 3**, which indicates that a combination of activating substances can further enhance the pro-inflammatory response.

The same experiments were also carried out on ivory slices and essentially showed similar results. However, the overall expression of IL-8 was much higher, which could be due to different conditions on ivory slices or 6-well dishes instead of 24-well dishes, for example. That is why these experiments were not combined in statistical calculations. Nevertheless, the relative increase of IL-8 was the same under both experimental settings.

**Figure 4:**
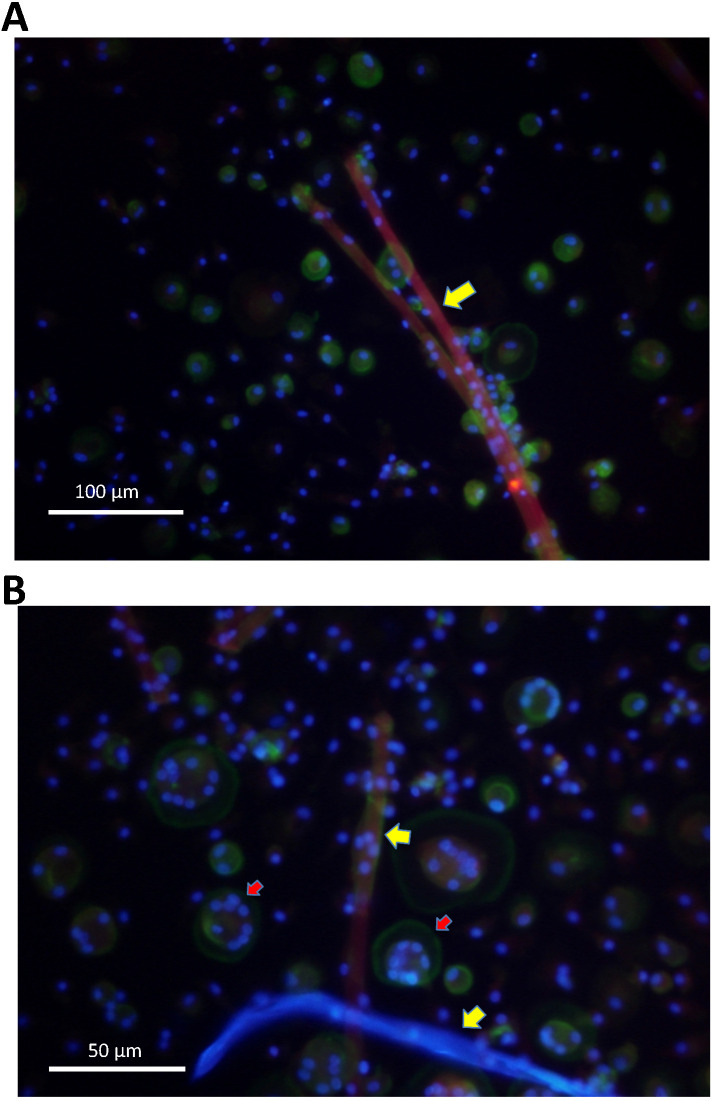
** A.** Monocytes were isolated from peripheral blood and stimulated with surgical suture material (red). After 16 days multinucleated giant cells had formed in direct vicinity to surgical suture material (yellow arrows), as demonstrated by fluorescence microscopy. **B.** Cells that were in direct vicinity to surgical suture material (yellow arrows) showed multiple nuclei (blue) and actin rings (geen colour; indicated by red arrows), which are typical for osteoclasts as demonstrated by fluorescence microscopy.

Further on, cells were evaluated by fluorescence microscopy. Giant cells attached to surgical suture material and showed multiple nuclei, as well as actin rings, known as typical features of osteoclasts (**Figure 4A,B**).

In order to evaluate functionality of these cells, similar experiments were performed on ivory slices and resorption pits were evaluated by two independent observers.

**Figure 5:**
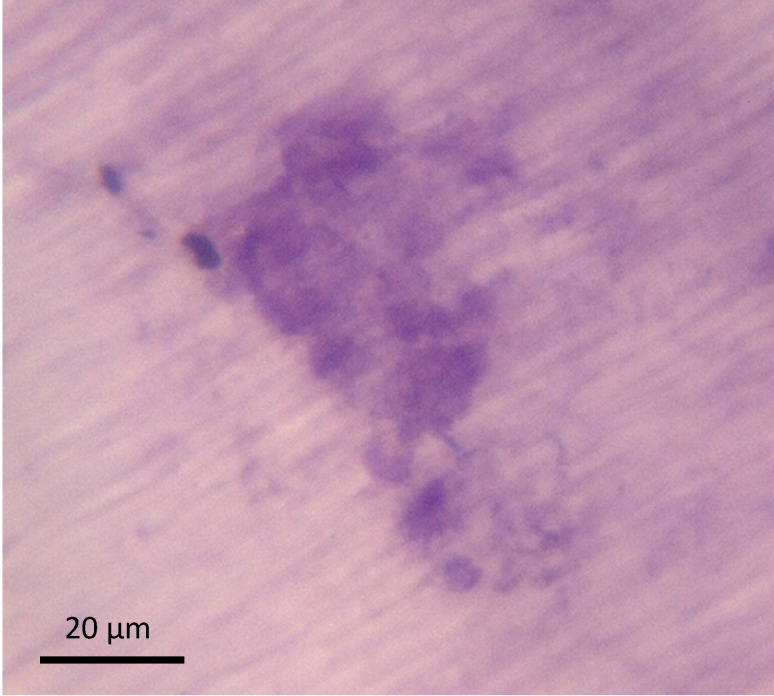
After incubation for 16 days, ivory slices were stained with toluidine blue and resorption pits were counted by two independent observers.

As shown in **Table 3**, more resorption pits were found after stimulation with LTA and with surgical suture material. The most resorption pits were seen after stimulation with LTA and suture material together, which again underlines the notion that a combination of stimuli can further enhance the inflammatory response (**Figure 5**).

**Figure 6:**
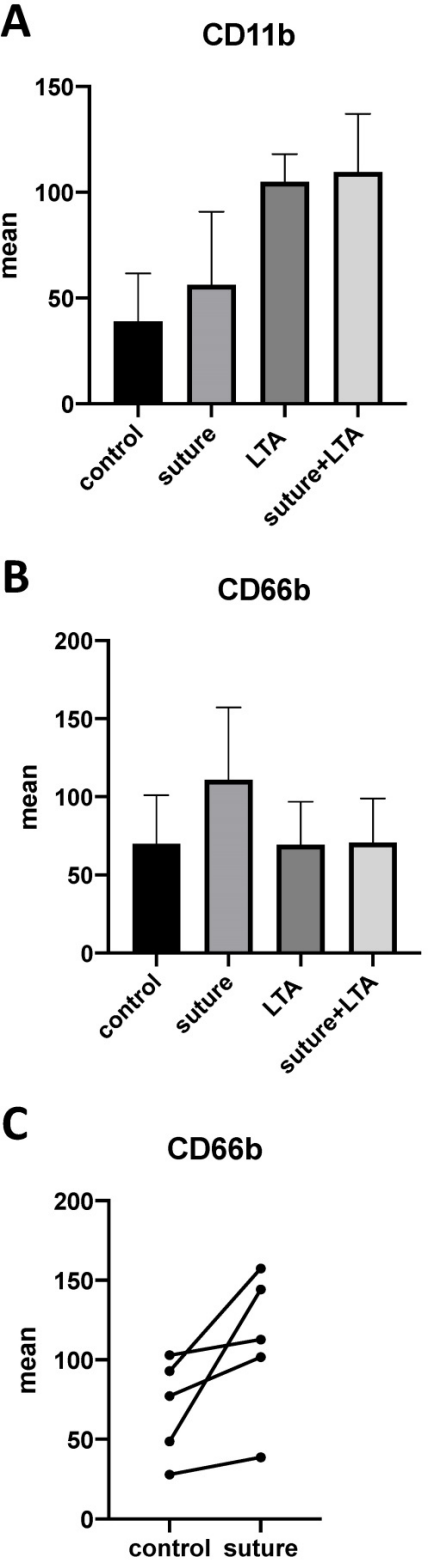
** A-C.** Whole blood samples were stimulated with surgical suture material and/or LTA. **A,B.** Up-regulation of the activation-associated cell surface markers CD11b and CD66b could be observed by FACS (fluorescence-activated cell sorting) analysis. **C.** Individual response among different donors varied greatly, but an up-regulation could be seen for each donor. The data for 5 individuals are shown.

In our previous work we were able to demonstrate that polymorphonuclear neutrophils (PMN) are predominant in implant-associated infections and that these cells release pro-inflammatory cytokines, such as MRP-14 and IL-8, which leads to osteoclast generation. Therefore, we were interested in evaluating whether PMNs were also affected by surgical suture material. After stimulating whole blood samples with suture material and LTA, we were able to demonstrate an increase of the activation-associated cell surface markers CD11b and CD66b on neutrophils (**Figure 6A-C**). Although each donor showed an enhanced response after stimulation with surgical suture material, the individual response varied greatly between different donors, and therefore statistical significance was not reached (**Figure 6C**).

## Discussion

In our previous work, we were interested in evaluating the pro-inflammatory environment in implant-associated infections and aseptic implant loosening, particularly with regard to bone resorption. Similar local defence mechanisms are activated, although the number and composition of the cellular infiltrate vary 11. In both cases, phagocytosis of either bacteria or of wear particles is initiated by monocytic cells and in implant-associated infections also by neutrophils. Phagocytosis is associated with a release of various cytokines which results in the generation of a pro-inflammatory environment. As the inflammatory response progresses, monocytes differentiate into osteoclasts, the latter being the only cell type capable of degrading bone. Because the initial insult persists (phagocytosis of bacteria or wear particles), so does the inflammatory response, which eventually leads to osteolysis and further on implant loosening 4, 9, 10, 12, 26.

We evaluated tissue samples that were collected intra-operatively from patients suffering from implant-associated infections. Histopatholgical analysis revealed that multinucleated giant cells were detected in areas of osteolysis. Due to their positivity for the specific protease cathepsin K, they could be identified as osteoclasts.

However, multinuclear giant cells were not merely distributed along bone substance. We also observed a conspicuous number of giant cells in direct vicinity to surgical suture material. The fact that a “foreign body”, such as calcifications, wear particles, uremic cristals, or indeed surgical suture material can induce a monocytic immune reaction with generation of foreign body macrophages, has been known, however whether such type of immune reaction can also lead to osteoclastogenesis was still unknown^4^, ^21^. Therefore, we were interested whether these cells are in fact also functional osteoclasts.

The formation of multinuclear giant cells in response to surgical suture material has been described in literature. Lovric et al. used a murine synovial airpouch model to demonstrate formation of multinuclear giant cells around various suture materials, as well as an enhanced expression of matrix metalloproteinases (MMP) -1,-2,-9, and -13 in particular when suture wear particles were compared to intact sutures 18. Lambertz et al. evaluated the response to 6 different surgical suture materials by histopathology in a rat model. They described a foreign body granuloma and a comet tail-like infiltrate (CTI) for each suture material, however the CTI was particularly enhanced when Vicryl (polyglactin 910) was used 22.

An *in vitro* study by Lock et al. using human monocyte THP-1 cells showed similar results concerning Vicryl sutures. Enhanced gene expression of the pro-inflammatory cytokine IL-8, as well as a decrease of the anti-inflammatory cytokine TGFβ1 was observed 23.

Moreover, there are also clinical reports available, which describe that revision surgeries were performed due to suspected post-operative infections, but histopathological evaluation of tissue samples revealed granulomatous inflammation due to suture material (Vicryl) instead 27, 28.

Vicryl is an absorbable suture which is composed of 90% glycolide and 10% L-lactide and is widely used for closure of deep fascial and subcutaneous layers 29.

In this study, we also used Vicryl polyglactin 910 sutures for our *in vitro* experiments, because this type of surgical suture material was used in all patients.

In order to prove that multinucleated giant cells in direct vicinity to surgical suture material were also functional osteoclasts, immunohistochemical staining for cathepsin K was performed. Cathepsin K is a protease which is involved in bone resorption and has been described as typical for osteoclasts 30-32.

As shown in **Figure 2**, multinucleated giant cells were indeed positive for cathepsin K and therefore follow-up *in vitro* experiments were performed.

We were able to show by fluorescence microscopy that human monocytes differentiated into osteoclasts when stimulated with surgical suture material. Cells showed multiple nuclei, as well as the osteoclast-typical actin ring. Functionality of osteoclasts was tested by performing the experiments on ivory slices. Resorption pits were evaluated and higher numbers were counted after stimulation with surgical suture material. Furthermore, cell supernatant samples were collected and increased release of IL-8 was observed. Interleukin-8 is a well-established pro-inflammatory cytokine and has been previously linked to osteoclast generation. Similarly, Lock et al. also demonstrated in their study that IL-8 gene expression in monocyte THP-1 cells was enhanced following stimulation with Vicryl sutures 23.

Because the cellular infiltrate in implant-associated infections comprises not merely macrophages but also high numbers of neutrophils, we were interested in the effect of surgical suture material on these cells. Our results show that the activation-associated cell surface markers CD11b and CD66b were up-regulated after stimulation with surgical suture material, suggesting that not only macrophages but also neutrophils recognize and respond to suture material. As previously reported in literature, the infiltration of neutrophils in chronic inflammatory diseases correlates with the number of osteoclasts found in tissue samples 33. Activated neutrophils might also directly stimulate osteoclastogenesis via release of IL-8, therefore linking inflammation and bone erosion.

Additionally, we were interested in evaluating whether a combination of different stimuli could further enhance the pro-inflammatory response. Therefore, minor amounts of LTA -which is a major component of the bacterial cell wall of gram-positive bacteria-, to mimic infection, was added. We were able to show that the numbers of resorption pits as well as the release of IL-8 were further increased when human monocytes were stimulated with minor amounts of LTA and surgical suture material together.

The additional effect that minor amounts of bacterial products or endotoxins might have on immune cells has been well-described in particular in regard to aseptic implant-loosening. Bi et al. were able to show *in vitro* and *in vivo* that adherent endotoxin on titanium wear particles enhanced pro-inflammatory cytokine production and osteoclast differentiation 17 and their *in vivo* experiments demonstrated that particle-induced osteolysis was reduced by 50-70% if adherent endotoxin was removed.

Our experiments indicate that bacterial components and surgical suture materials might also elicit a synergistic effect on immune cells, which leads to increased osteoclast generation.

These findings might not only be relevant in the context of orthopaedic implant-associated infections. It has been previously described in literature that macrophages release a variety of products including proteases after induction by phagocytosis and/or inflammatory stimuli. The release might be enhanced, particularly if phagocytosis of large particles is unsuccessful and has been described to contribute to tissue damage in chronic inflammatory disease such as rheumatoid arthritis 34, 35.

Impaired phagocytosis by macrophages might also have effects on tissue repair processes and could result in severe post-surgical complications such as anastomotic leakage 36.

## Conclusions

Implant-associated infections are difficult to treat and often require multiple revision surgeries due to persistent infection. Our results show that in these cases, the immune system is constantly activated not only by bacterial components but also by surgical suture material which is renewed during every revision surgery. We were able to demonstrate for the first time, that the generation of functional osteoclasts can also be induced by surgical suture material and is aggravated under septic conditions, which further contributes to implant-loosening. This is a further step to understand implant loosing after joint replacement.

## Figures and Tables

**Table 1:** ** Patients' clinical data.** Five patients with infectious implant loosening who underwent removal of the implant were included in the study. Microbiological evaluation of tissue samples revealed multiple bacteria species in one patient

Characteristics	
**Gender**	
Male	n=3
Female	n=2
**Age (years)**	
Mean (±standard deviation)	68 (±18.96)
Median (range)	72 (41-91)
**Loosening of total joint replacement**	
Hip	n=1
Knee	n=4
**CRP (C-reactive protein) concentration**	
Mean (±standard deviation)	116.9 (±113.7) mg/L
**WBC (white blood cell count)**	
Mean (±standard deviation)	8.4 (±1.4) /nl
**Bacteria species detected by culture of tissue samples**	
*Enterococcus faecalis*	n=1
*Staphylococcus aureus*	n=3
*Coagulase-negative Staphylococci*	n=2

**Table 2:** IL-8 (pg/ml) measured in cell supernatant of monocytes after 48 hours, 7 days and 14 days of incubation. Differences between medium (control) and groups were calculated using unpaired *t*-test with *p*<0.05. Experiments were repeated twice and additionally also on ivory slices

	Medium IL-8(pg/ml)	LTA IL-8(pg/ml)	Surgical suture material IL-8(pg/ml)	LTA+surgical suture material IL-8 (pg/ml)	RANKL+M-CSF (positive control) IL-8 (pg/ml)
48 h	61,7	497,3	101,6	890	242,4
48 h	144,3	2224,8	127	1793,9	550,4
7 d	175,5	1319,5* (*p*=0.0393)	369,6* (*p*=0.0127)	4688	974,6* (*p*=0.0156)
7 d	129,7	1916,2*	355,7*	2362,8	795,2*
14 d	98,6	281,6	237,8	483,9	217,3
14 d	72,1	2312,3	80,4	3191,5	186,2

**Table 3:** Resorption pits were evaluated by two independent observers. Stimulation with LTA and surgical suture material in particular showed the highest numbers of resorption pits. Experiments were carried out multiple times. Results of one experiment are shown

	Number of resorption pits, observer 1	Number of resorption pits, observer 2
Medium (control)	n=0	n=0
LTA	n=81	n=70
Surgical suture material	n=139	n=110
LTA+surgical suture material	n=201	n=166
RANKL/M-CSF (positive control)	n=59	n=50
